# Mesenchymal Stem Cells and Extracellular Vesicles Derived from Canine Adipose Tissue Ameliorates Inflammation, Skin Barrier Function and Pruritus by Reducing JAK/STAT Signaling in Atopic Dermatitis

**DOI:** 10.3390/ijms23094868

**Published:** 2022-04-27

**Authors:** Sung Youl Kim, Tae Hong Yoon, Jungtae Na, Seong Joon Yi, Yunseok Jin, Minji Kim, Tae-Ho Oh, Tae-Wook Chung

**Affiliations:** 1 GNG CELL Co., Ltd., R&D Center, 122 Unjung-ro, Bundang-gu, Seongnam-si 13466, Korea; ks10204@naver.com (S.Y.K.); enteryth@naver.com (T.H.Y.); 2 Department of Life Science, Sogang University, 35 Baekbeom-ro, Mapo-gu, Seoul 04107, Korea; pugokjebi@gmail.com; 3 Department of Veterinary Anatomy, College of Veterinary Medicine, Kyungpook National University, 80 Daehak-ro, Buk-gu, Daegu 41566, Korea; sjyi@knu.ac.kr; 4 Department of Veterinary Internal Medicine, College of Veterinary Medicine, Kyungpook National University, 80 Daehak-ro, Buk-gu, Daegu 41566, Korea; jinyunseok@naver.com (Y.J.); kmg532@hanmail.net (M.K.); 5 JIN BioCell Co., Ltd., R&D Center, #101-103, National Clinical Research Center for Korean Medicine, Pusan National University Korean Medicine Hospital, 20 Geumo-ro, Mulgeum-eup, Yangsan-si 50612, Korea

**Keywords:** atopic dermatitis, canine, mesenchymal stem cell, extracellular vesicle, inflammation, skin barrier function, pruritus

## Abstract

Canine atopic dermatitis (AD) is a common chronic inflammatory skin disorder resulting from imbalance between T lymphocytes. Current canine AD treatments use immunomodulatory drugs, but some of the dogs have limitations that do not respond to standard treatment, or relapse after a period of time. Thus, the purpose of this study was to evaluate the immunomodulatory effect of mesenchymal stem cells derived from canine adipose tissue (cASCs) and cASCs-derived extracellular vesicles (cASC-EVs) on AD. First, we isolated and characterized cASCs and cASCs-EVs to use for the improvement of canine atopic dermatitis. Here, we investigated the effect of cASCs or cASC-EVs on DNCB-induced AD in mice, before using for canine AD. Interestingly, we found that cASCs and cASC-EVs improved AD-like dermatitis, and markedly decreased levels of serum IgE, (49.6%, *p* = 0.002 and 32.1%, *p* = 0.016 respectively) epidermal inflammatory cytokines and chemokines, such as IL-4 (32%, *p* = 0.197 and 44%, *p* = 0.094 respectively), IL-13 (47.4%, *p* = 0.163, and 50.0%, *p* = 0.039 respectively), IL-31 (64.3%, *p* = 0.030 and 76.2%, *p* = 0.016 respectively), RANTES (66.7%, *p* = 0.002 and 55.6%, *p* = 0.007) and TARC (64%, *p* = 0.016 and 86%, *p* = 0.010 respectively). In addition, cASCs or cASC-EVs promoted skin barrier repair by restoring transepidermal water loss, enhancing stratum corneum hydration and upregulating the expression levels of epidermal differentiation proteins. Moreover, cASCs or cASC-EVs reduced IL-31/TRPA1-mediated pruritus and activation of JAK/STAT signaling pathway. Taken together, these results suggest the potential of cASCs or cASC-EVs for the treatment of chronic inflammation and damaged skin barrier in AD or canine AD.

## 1. Introduction

Mesenchymal stem cells (MSCs) are cells with self-renewal and multipotency. MSCs reside in various tissues, such as bone marrow, adipose, umbilical cord and kidney [1], and can differentiate in osteoblasts, adipocytes, myocytes and chondrocytes [2,3,4,5]. Since the establishment of MSCs, cell therapy methods using MSCs have been reported in various disease models, such as autoimmune encephalomyelitis [6], myocarditis [7], colitis and sepsis [8], and glomerulonephritis [9]. In addition, MSCs have immunomodulatory abilities to regulate the suppression of Th2 cells and increase regulatory T (Treg) cells [10,11]. In particular, MSCs have no major histocompatibility complex (MHC) II and co-stimulatory molecules, such as CD40, CD80 and CD86, play a major role in allogeneic antigen recognition, and have low MHC I expression [12]. Thus, the therapeutic effect of using immunomodulatory action can be clinically expected, because immunogenicity of MSCs is relatively low [13,14].

Recent studies have shown that MSCs exert an immunosuppressive effect by generating and releasing extracellular vesicles (EVs) of various sizes composed of lipid bilayers, rather than by the effect of cell-cell contact [15]. EVs include exosomes (a diameter of 40–150 nm) [16] and micro-vesicles (a diameter of 100–1000 nm) [17]. EVs are considered essential mediators of cellular communication encapsulating various genetic materials. In addition, MSC-derived EVs (MSC-EVs) contain regulatory molecules that can regulate immune cell function [18,19]. MSC-EVs have also been shown to have immunomodulatory abilities similar to those seen in MSCs [19].

Atopic dermatitis (AD) in dogs is an allergic skin disease with a prevalence of about 10–15% and has many similarities to human AD [20,21]. Although the cause of canine AD is not clear, it is known to alter the skin barrier and the immunological response as the result of complex interactions, such as genetic factors (filaggrin mutation), environmental factors (allergens) and imbalance of immune function [21,22,23,24,25,26]. Current canine AD treatments use immunomodulatory drugs, but some of the dogs have limitations that do not respond to standard treatment, or relapse after a period of time [20,27,28]. Furthermore, because the long-term use of anti-histamines, steroids etc. results in serious side effects, along with increased drug-resistance, there is a need to develop a drug that is both safe and fundamentally able to cure [27,28]. Thus, the development of various treatments, such as antibody and stem cell therapy, is required in treating canine AD.

Canine-derived MSCs have been isolated from various tissues, such as bone marrow, adipose tissue, muscle and periosteum [29]. Among these, MSCs derived from adipose tissue can differentiate into adipogenic, chondrogenic, myogenic and osteogenic cells like human MSCs [30]. Although there are currently many studies on both animals and humans demonstrating the efficacy of human MSCs in AD [31,32], few studies have applied canine MSCs to treat AD. Furthermore, there is no study in efficacy of cASC-EVs on AD. Thus, the purpose of this study was to investigate the immunomodulatory functions of cASCs and cASC-EVs in a DNCB-induced AD mice model to confirm their applicability to canines.

## 2. Results

### 2.1. Characterization of cASCs and cASC-EVs

**Figure 1:**
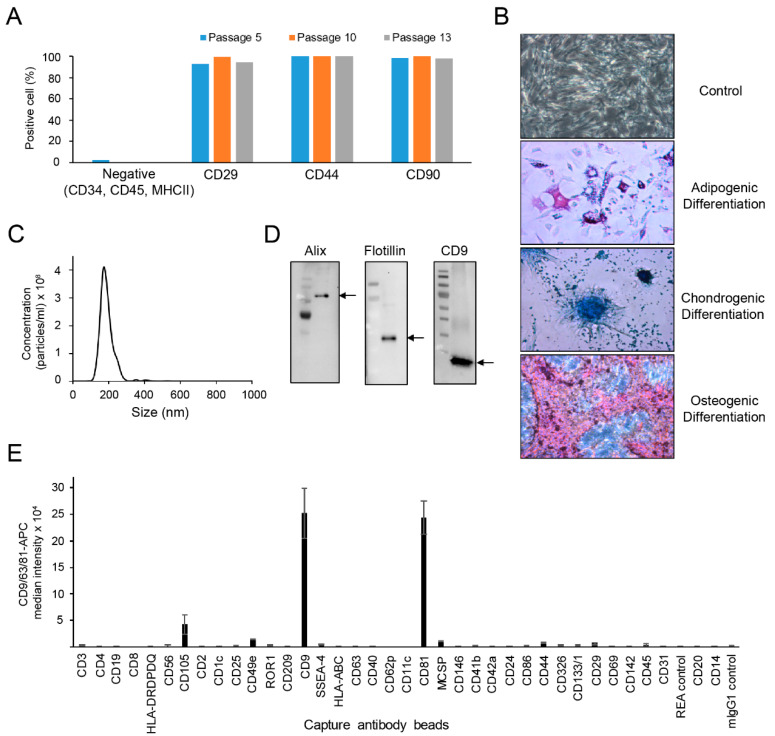
**Characterization of canine adipose tissue-derived mesenchymal stem cells (cASC) and cASC-EV.** (**A**) Bar graph obtained from flow cytometry data show the expression of MSC-positive markers such as CD29, CD44 and CD90 in cASCs populations. (**B**) Representative data showing the differentiation phenotypes of cASC. (**C**) Representative histogram of particle concentration and size distribution of cASC-EV measured by nanoparticle tracking analysis (NTA). (**D**) The presence of EV markers such as Alix, Flotilin and CD9 in cASC-EV as determined by Western blots. (**E**) Surface signature of cASC-EV quantified by MACSPlex EV kit in conjunction with flow cytometry. Data indicate APC median signal intensities of ASC-EV incubated with the 39 capture beads and stained with a mixture of CD9-, CD63-, and CD81-APC antibodies. Background was corrected by subtraction of median fluorescence APC intensity.

The profiles of cASCs revealed a homogeneous population of cells, positive for mesenchymal markers, such as CD29, CD44, and CD90 (Figure 1A). As expected, the isolated cASCs were successfully differentiated into adipocytes, chondrocytes or osteocytes with characterization of stem cells that can differentiate into each cell phenotype (Figure 1B). Next, we investigated the size distribution and concentration of cASC-EV particles using NTA. The expected exosomal size of cASCs is around 182 nm (Figure 1C). To determine the characterization of cASC-EVs, the exosomal specific markers were identified by western blot analysis. As shown Figure 1D, Alix, flotillin and CD9, as the exosomal specific markers, were clearly detected in the isolated cASC-EVs. In addition, we confirmed that several surface CD molecules, including CD105, CD49e, CD9, CD41b, CD81, CD44, and CD29, were markedly expressed in cASC-EV, but not in human major histocompatibility complex, such as human leukocyte antigen (HLA-DRDPDQ and HLA-ABC) and co-stimulatory molecules, such as CD80 and CD86 [33] (Figure 1E). These results suggest that the low immunogenicity of cASCs and cASC-EVs allow their allogeneic use for autoimmune diseases.

### 2.2. cASCs and cASC-EVs Alleviate Atopic Dermatitis Induced by DNCB Treatment

**Figure 2:**
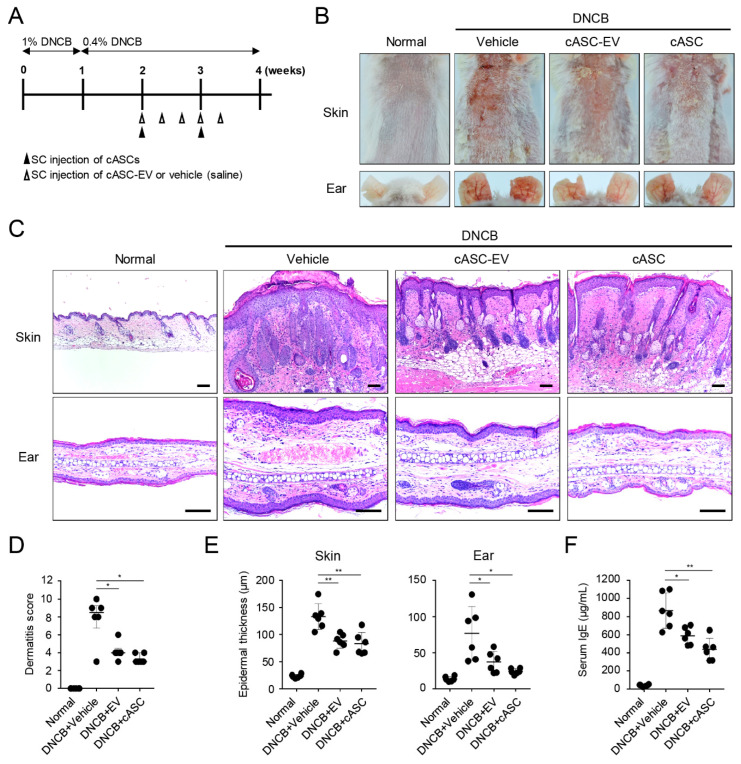
**cASC and cASC-EV improve DNCB-induced atopic dermatitis-like skin lesion in Balb/c mice.** (**A**) Schematic diagram for development of atopic dermatitis model by topical application of DNCB in Balb/c mice. After mice were sensitized with DNCB for 7 days, DNCB was further topically applied to the shaved dorsal skin and ear for 3 weeks. cASC was applied twice and cASC-EV was applied 5 times for 2 weeks (*n* = 6/group). (**B**) Representative dorsal skin and ear photographs of each treatment group showing comparison of AD-like skin lesions. (**C**) Tissue sections from the back skin and ear stained with hematoxylin and eosin (H&E). Scale bar, 100 μm. (**D**) The severity of dermatitis evaluated by a 3-point scoring index of atopic dermatitis. Dermatitis score was graded as 0 (absent), 1 (mild), 2 (moderate), or 3 (severe) based on the sum of the scores of clinical signs such as excoriation/erosion, scaling/dryness, edema, and erythema/hemorrhage. (**E**) Epidermal thickness of back skin and ear (*n* = 6/group). (**F**) The concentration of the serum total IgE levels measured by an ELISA. Dermatitis score represent median [IQR] (min–max) and the others represent mean ± SD. Significant value was * *p* < 0.05, ** *p* < 0.01 vs. DNCB + Vehicle.

To confirm the therapeutic potential of cASCs and cASC-EVs on an in vivo model, we established an atopic dermatitis (AD) mouse model induced by topical DNCB treatment (Figure 2A). AD-like skin lesions by DNCB showed skin severity with excoriation, scaling, edema, and erythema. Whereas, cASCs and cASC-EVs treatment alleviated DNCB-induced dermatitis compared to vehicle treatment (Figure 2B,D, Table 1 and Table 2). In addition, cASCs and cASC-EVs treatment resulted in suppression of epidermis hyperplasia, parakeratosis, and dermal edema induced by DNCB (Figure 2C,E and Table 1). Repeated treatment of topical DNCB remarkably increased serum IgE production, whereas administration of cASCs and cASC-EVs reduced levels of serum IgE as a major characteristic of AD (Figure 2F and Table 1).

### 2.3. cASCs and cASC-EVs Ameliorated Skin Inflammation in DNCB-Induced Atopic Dermatitis Mice

**Figure 3:**
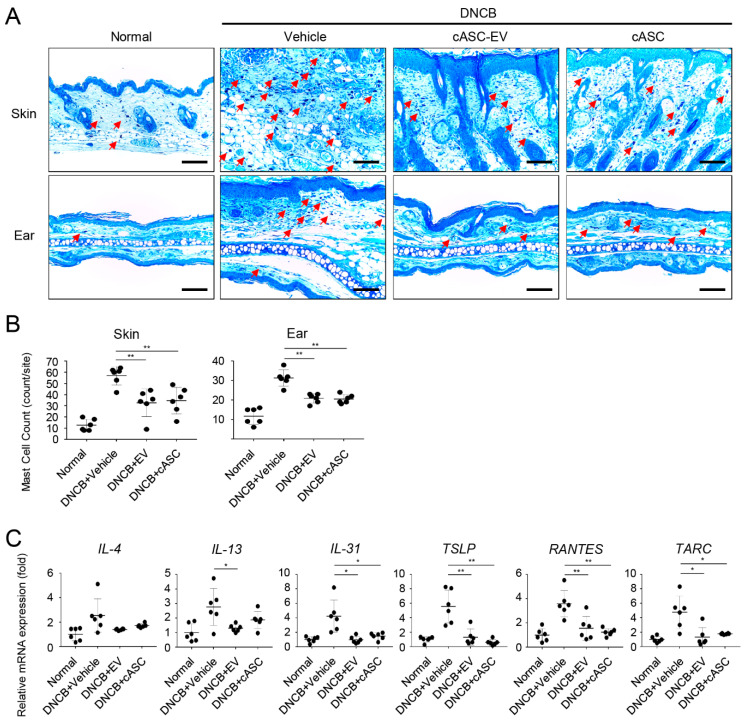
cASC and cASC-EV reduce the level of multiple inflammatory cytokines in atopic dermatitis-like skin lesions of Balb/c mice. (**A**) Tissue sections from the back skin and ears stained with Toluidine blue (TB). Red arrow indicates mast cell infiltrated into skin or ear tissue. Scale bar, 100 μm. (**B**) The number of mast cells counted from five random section areas (*n* = 6/group). (**C**) The expression of IL-4, IL-13, IL31, TSLP, RANTES and TARC genes on skin tissues as determined using real-time PCR (*n* = 6/group). The expression of TARC gene represent median [IQR] (min–max) and the others represent mean ± SD. Significant value was * *p* < 0.05, ** *p* < 0.01 vs. DNCB + Vehicle.

We further focused on determining local infiltration by mast cells. It is well known that histamine released by activated mast cells in a region of atopic dermatitis increases the permeability of blood vessels, causing immune cells to penetrate into tissues [34]. Thus, it is necessary to reduce activated mast cells to decrease allergic reactions like AD. cASC and cASC-EV administration markedly decreased the number of mast cells compared to the vehicle group on the dorsal skin and ears of mice treated with DNCB (Figure 3A,B and Table 1). In addition, we examined cytokine and chemokine productions in dorsal skin on day 28. The levels of the Th2 cytokines and chemokines, including IL-4, IL-13, IL-31, TSLP, RANTES and TARC, were enhanced by DNCB, while administration of cASC and cASC-EV reduced the levels of IL-4, IL-13, IL-31, TSLP, RANTES, and TARC in dorsal skin treated with DNCB (Figure 3C, Table 1 and Table 2). These results demonstrate that cASC and cASC-EV suppress the immune response of AD by regulating mast cell infiltration and the production of Th2-related cytokines and chemokines.

### 2.4. cASCs and cASC-EVs Promote Skin Barrier Repair in DNCB-Induced Atopic Dermatitis Mice

**Figure 4:**
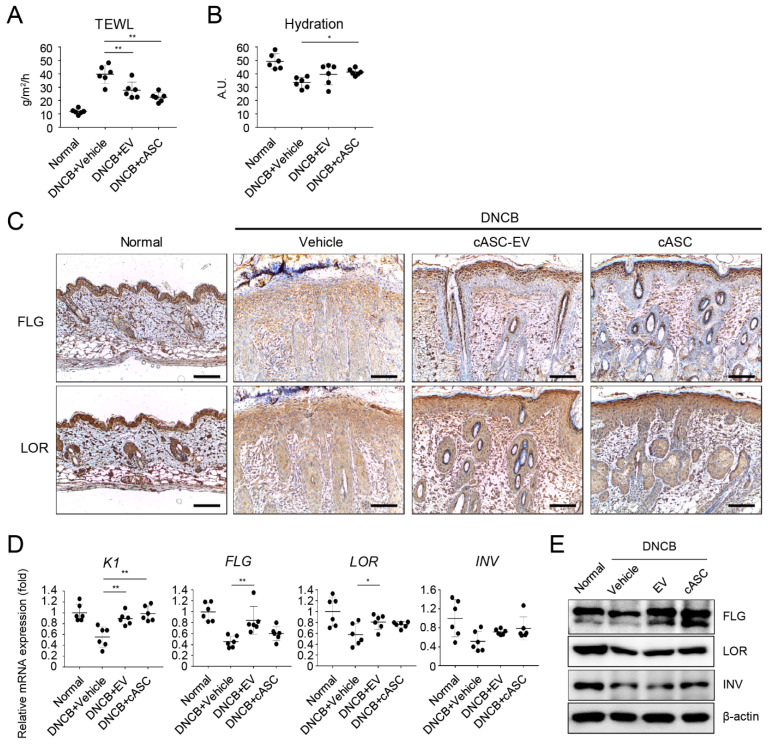
**cASC and cASC-EV improve epidermal barrier function in atopic dermatitis-like skin lesion of Balb/c mice.** (**A**) TEWL and (**B**) hydration of the AD-like skin lesions (*n* = 6/group). (**C**) Expression of filaggrin and loricrin as evaluated by immunohistochemical stain. Scale bar, 100 μm. (**D**) Transcripts of keratin 1, filaggrin, loricrin, and involucrin were quantified using real-time PCR (*n* = 6/group). (**E**) Immunoblots with anti-filaggrin, anti-loricrin, anti-involucrin, and anti-β-actin antibodies using lysates from skin. All data represent mean ± SD. Significant value was * *p* < 0.05, ** *p* < 0.01 vs. DNCB + Vehicle.

To evaluate skin barrier function, transepidermal water loss (TEWL) and hydration were measured by using tewameter and corneometer in DNCB-induced AD-like skin lesions. As shown in Figure 4A, multiple DNCB-exposures markedly resulted in the increment of TEWL and decrement of hydration on dorsal skin. However, increased TEWL of AD-like skin lesion markedly decreased in cASCs and cASC-EVs groups on day 28 compared to the vehicle group (Figure 4A and Table 1). Moreover, cASCs and cASC-EVs significantly recovered reduced hydration compared to the vehicle group (Figure 4B and Table 1). We also confirmed whether cASCs and cASC-EVs affect the expression levels of epidermal differentiation proteins, such as keratin1 (K1), filaggrin (FLG), loricrin (LOR), and involucrin (INV), to improve AD in dorsal skin lesions treated with DNCB. The expression of keratin1, filaggrin, loricrin, and involucrin significantly decreased in DNCB-induced AD-like skin lesions, which markedly improved following administration of cASCs and cASC-EVs (Figure 4C,E and Table 1). Collectively, these results indicate that cASCs and cASC-EVs might be effective in accelerating skin barrier repair.

### 2.5. cASCs and cASC-EVs Suppressed IL-31-Mediated Pruritus and Phosphorylated STATs in DNCB-Induced Atopic Dermatitis Mice

**Figure 5:**
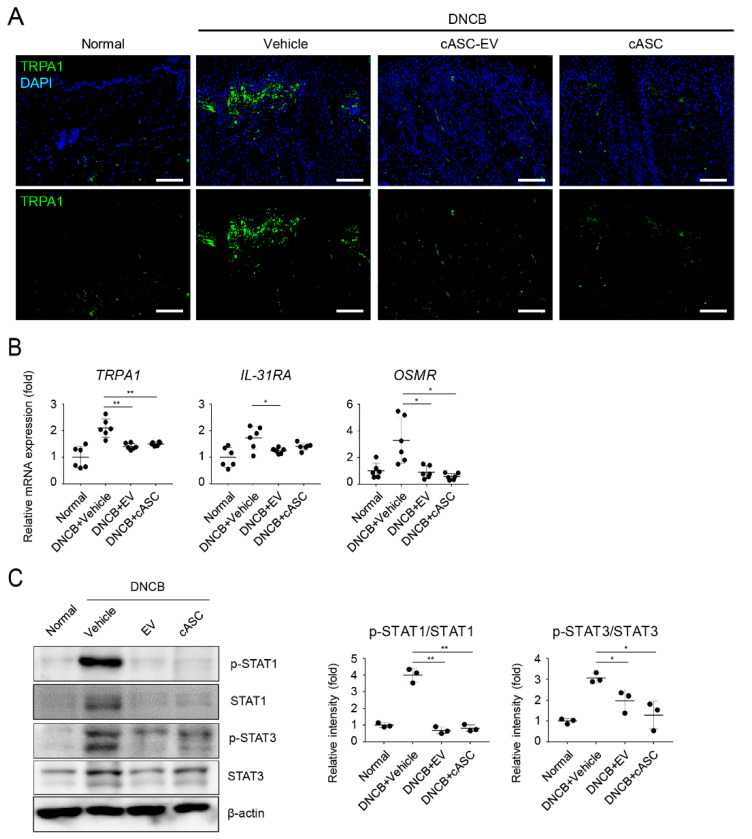
cASC and cASC-EV inhibit pruritogen responses through downregulation of phosphorylation of STATs in atopic dermatitis-like skin lesion of Balb/c mice. (**A**) Tissue sections from back skins stained with TRPA1 using Immunofluorescence. Scal bar = 100 μm. (**B**) The expression of TRPA1, OSMR and IL-31RA genes in skin tissues; each group represented using real-time PCR (*n* = 6/group). (**C**) Immunoblots with anti-p-STAT1, anti-STAT1, anti-p-STAT3, anti-STAT3 or anti-β-actin antibodies using lysates from skin. Western blots were analyzed quantitatively. All data represent mean ± SD. Significant value was * *p* < 0.05, ** *p* < 0.01 vs. DNCB + Vehicle.

We then evaluated whether cASCs and cASC-EVs could decrease pruritus in DNCB-induced AD-like skin lesions. To understand the potential role of cASCs and cASC-EVs, we performed fluorescent IHC of TRPA1, activated under inflammatory conditions, on AD dorsal skin [35]. The expression of TRPA1 significantly increased in the epidermal and dermal regions of DNCB-induced AD (Figure 5A and Table 1). Conversely, the expression of TRPA1 was rare in cASC and cASC-EV-administered skin lesions (Figure 5A and Table 1). Our previous data showed that the expression of IL-13 induced by DNCB was decreased by the administration of cASCs and cASC-EVs (Figure 3C and Table 1). It has been reported that IL-13 highly stimulates TRPA1 expression in mast cells [36] and IL-31-induced pruritus decreased in TRPA1-deficient mice [37]. Additionally, cASC and cASC-EVs markedly inhibited the expression of IL-31 receptor A (IL-31RA) and the Oncostatin M Receptor (OSMR) known as IL-31 receptors (Figure 5B and Table 1) as well as IL-31 (Figure 3C and Table 1). Several reports have shown that IL-4, IL-13, and IL-31 induces the expression of specific genes by activating STATs following activation of Janus-kinses (JAKs) [38,39]. To evaluate the regulation of JAK-STATs signaling by cASCs and cASC-EVs in DNCB-induced AD like lesions, we investigated the activation of STAT1 and STAT3 in dorsal skin. cASC and cASC-EVs markedly decreased levels of phosphorylated STAT1 and STAT3 induced by DNCB. These results suggest that increase of Th2-related cytokines and IL-31-induced TRPA1 expression are induced by STAT1 and STAT3 signaling, and cASC and cASC-EVs improve AD by suppressing STAT1 and STAT3 signaling.

## 3. Discussion

Mesenchymal stem cells have inflammatory therapeutic functions through the interaction of paracrine factors, such as extracellular vesicles, cytokines, chemokines, and growth factors, with immune cells. EVs secreted from MSCs contain various active molecules, such as bioactive proteins, lipids, mRNAs, and microRNAs. Numerous recent studies have reported that the immunomodulatory ability of hMSCs can be useful in the treatment of allergic diseases, such as asthma and dermatitis [40,41,42,43,44]. In addition, studies have been conducted on the immunosuppressive effects of EVs secreted by MSCs. MSC-EVs are considered a new alternative to reducing potential side effects, such as the uncontrolled differentiation and proliferation potential of MSCs [18,19,45,46]. Thus, many researchers are trying to research the immunological therapeutic application of MSCs and MSC-EVs to cure AD, which is a common inflammatory skin disease, resulting from skin barrier defects and immunological hyperactivity. However, there are few therapeutic studies of canine AD on cMSCs [47,48], and not yet any studies on the efficacy of cMSC-EVs for the treatment of canine AD. Here, MSCs were isolated from canine adipose tissue, and EVs were further isolated and characterized as shown in Figure 1. In addition, cASCs and cASC-EVs were subcutaneously injected into DNCB-induced AD in mice to analyze their immunomodulatory ability, before using them for canine AD. Subcutaneous administration of cASC led to a high level of therapeutic efficacy. Interestingly, the application of cASC-EVs showed a similar therapeutic efficacy compared to cASC administration. In this study, we demonstrated that cASCs and cASC-EVs suppress DNCB-induced AD in mice, probably through the inhibition of Th2 cytokine and chemokines, causing improvement of skin barrier function and pruritus.

Canine AD results in various factors, including a large amount of IgE, T lymphocyte polarization, and mast cell hyperplasia [49]. Furthermore, canine AD disease typically begins with Th2 cells, and progresses to a chronic disease with a mixed Th2/Th1 profiles [50,51]. Schlotter et al. reported that Th2 cells prominently distributed in the acute skin lesions of canine AD produce cytokines, such as interleukin (IL)-4, IL-13 and IL-31 [23]. In chronic AD, IL-2, IFN-γ and IL-18 secreted from Th1 cells are detected [24,50]. IL-4 and IL-13 are involved in the proliferation and differentiation of B cells, and promote the production of IgE antibodies, by activating the infiltration of eosinophils to the site of inflammation [24,50]. In addition, IL-31 is actively involved in pruritus [51,52]. It seems that the cytokine pathway of canine AD is very similar to that of human AD [53,54,55]. According to a previous study, 22 of 26 AD dogs administered allogeneic canine adipose MSCs (cAd-MSC) were treated without systemic immunosuppressant medication at follow-up of 6 months [48]. None of the dogs showed systemic local adverse reactions after administration of allogeneic cAd-MSC [48]. Moreover, several studies have proven that injected MSCs migrate to damaged and inflammatory tissues, resulting in a therapeutic effect on the diseases [32,56,57,58]. In AD, MSCs migrate to skin lesions through draining lymph nodes and reduce cell infiltration, such as T cells in skin lesions [32]. In the present study, we also observed infiltration of mast cells, and higher expression of IL-4, IL-13, IL-31, TSLP, RANTES and TARC in AD-like region of mice treated with DNCB. On the other hand, the administration of cASC and cASC-EVs reduced mast cell infiltration and the production of Th2-related cytokines and chemokines (Figure 3). Moreover, the enhanced levels of serum IgE in DNCB-induced AD mice was markedly decreased by cASC and cASC-Evs (Figure 2). These results indicated that cASCs and cASC-EVs inhibited skin inflammation in DNCB-induced AD mice through modulation of inflammatory circumstance, mediated by activation of T cells and mast cells.

AD is a pruritic skin disorder with barrier dysfunction and elevated expression of IL-4 and IL-13. In addition, the barrier dysfunction is correlated with the downregulation of barrier-related molecules, including K1, FLG, LOR and IVL. IL-4 and IL-13 potently suppress the expression of barrier-related molecules which play crucial roles in maintaining the structural integrity and functions of stratum corneum, and help in maintaining the homeostasis of stratum corneum [59,60]. The expression and release of IL-31 induced by IL-4 in Th2 cells are a recently identified cytokine with a well-defined role in the pathogenesis of pruritus. Interleukin-31 activates the JAK1/JAK2 and STAT3 (also to less extent STAT1 and STAT5) pathways via IL-31R which is a heterodimer composed of IL-31RA and OSMR [61,62]. In this study, we confirmed the downregulation of K1, FLG, LOR and IVL in AD-like regions of mice treated with DNCB (Figure 4). Furthermore, IL-31/IL-31R was upregulated in AD-like regions. However, cASCs and cASC-EVs clearly recovered the downregulated barrier-related genes and proteins as well as the upregulated IL-31/IL-31R expression. In addition, STAT1 and 3 activations in AD-like regions were significantly suppressed by cASCs and cASC-EVs (Figure 5). Taken together, for improvement of AD, cASCs and cASC-EVs regulated histamine or IgE release by prohibiting infiltration of mast cells, and skin barrier dysfunction induced by IL-4 and IL-13 by blocking infiltration of T cells, and pruritus skin stimulated by the phosphorylation of STAT1, and 3 through the increment of IL-31/IL-31R expression in T cell by IL-4. However, using 6 mice per group in this study has shown limitation in the significance of some data in statistical analysis, although there is tendency for positive effects of cASCs and cASC-EVs on AD-skin.

Similar immunomodulatory effects of cASC-EVs, compared to cASCs, on AD were demonstrated in this study. MSC-EVs have been studied in various disease models [63,64]. Although clinical trial results for human-derived MSCs confirmed the therapeutic efficacy of human-MSCs [65], so far, there is no direct evidence of the clinical relevance of canines to exosomes derived from canines. The current study provides a basis for the treatment of AD in canines as well as humans, as it has been known that the paracrine factors, containing EVs, are one of the therapeutic effects of MSCs [18,19].

## 4. Materials and Methods

### 4.1. Isolation, Culture, and Characterization of cASC

The adipose tissue was obtained from healthy beagle dogs with the approval of the Ethics Committee for Experimental Animal Research at Kyungpook National University (IACUCSGU2019_15). ASCs were isolated from tissues and cultures as previous described [66,67]. Primary cultures were carried out with Dulbecco’s modified Eagle’s medium /F12 (cytiva, Logan, UT, USA) containing 10% fetal bovine serum (cytiva, Logan, UT, USA), 10 ng/mL epidermal growth factor (EGF, ProSpec, Rehovot, Israel), 2 ng/mL basic fibroblast growth factor (bFGF, PeproTech, Rocky Hill, NJ, USA). 10 ng/mL insulin-like growth factor (IGF, ProSpec, Rehovot, Israel), 2.5 mM L-glutamine (Gibco, Grand island, NY, USA) 100 U/mL penicillin and 100 µg/mL streptomycin (Gibco, Grand island, NY, USA). Cells were detached when confluence was over 80%, and sub-cultured at a concentration of 10^4^ cells/cm^2^ for continued passaging. The remaining cells were cryopreserved, and stored in liquid nitrogen. All experiments and in vivo implantation were conducted at passage 2. The cells were characterized for the expression of several stem cell markers by flow cytometry (Beckman Coulter, Brea, CA, USA) before they were used in the experiments.

### 4.2. Isolation and Characterization of cASC-EV

ASC-EV were isolated from the ASCs CM by the tangential flow filtration (TFF)-based method [46,68]. Briefly, the CM was filtrated through a 0.22-µm polyethersulfone membrane filter (Merck Millipore, Billerica, MA, USA) to remove non-ev particles, such as cells, cell debris, micro-vesicles and apoptotic bodies. The CM was then concentrated by tangential-flow filtration with a 500 kDa molecular weight cut-off filter membrane cartridge (Pall, Port Washington, NY, USA), and then buffer exchange was performed by diafiltration with PBS (WELGENE, Gyeongsan, Korea). Isolated ASC-EV were aliquoted into polypropylene disposable tubes, and stored at −80 °C until use. Before using, frozen ASC-EV were left at 4 °C until completely thawed and were not frozen again. The characterization and profile analysis of ASC-EV were conducted following the Minimal Information for Studies of Extracellular Vesicles 2018 (MISEV2018) recommended by the International Society of Extracellular Vesicles (ISEV) [15].

To determine size distribution and particle concentration, cASC-EV diluted with PBS were analyzed by nanoparticle tracking analysis (NTA) using a NanoSight LM10 (Malvern Panalytical, Worcestershire, UK) equipped with a 642-nm laser. cASC-EV, diluted with PBS to between 100 and 200 particles per frame, were scattered and illuminated by the laser beam and their movements under Brownian motion were captured for 60 s each at a camera level of 16. Videos were then analyzed by the NTA 3.2 software using constant settings. To provide a representative result, at least 5 videos were captured and >2000 validated tracks were analyzed for each individual sample. The NTA instrument was regularly checked with 100 nm-sized standard beads (Malvern Panalytical, Worcestershire, UK). To provide representative size distribution of particle, size distribution profiles from each video replicates were averaged.

### 4.3. Animal Care and Induction of AD Model

Six-week-old male BALB/c mice were supplied by DBL, Inc. (Incheon, Korea). Solid feed (no antibiotics added) and water were supplied until the day of the experiment, and the animals were used for experiments following a 1-week adaptation period at temperatures of 23 ± 2 °C, humidity of 55 ± 10% and a 12–12 h light–dark cycle. All animal experiments were conducted in accordance with the principles of laboratory animal care of the National Institutes of Health (NIH, Bethesda, MD, USA) and with the approval of the Ethics Committee for Experimental Animal Research at Sogang University (IACUCSGU2019_15).

Repeated application of 2,4-dinitrochlorobenzene (DNCB; Sigma-Aldrich, Merck KGaA, Darmstadt, Germany) to a local site causes AD-like skin lesions. In the first inflammation induction process, 100 μL of 1% DNCB solution (acetone:olive oil = 4:1) was applied to the same dorsal area twice for 3 days in all groups, except in the normal group (NOR). The vehicle (acetone:olive oil = 4:1) was used to treat the NOR group. Following a latent period of 4 days, 100 μL of 0.4% DNCB was used for a second sensitization. 8 h after every DNCB exposure, cASCs (2 × 10^6^ cells/head) or cASC-EVs (2 × 10^10^ particles/head) were subcutaneously applied twice or 5 times for 2 weeks, respectively. Vehicle (saline) served as a negative control.

Basal transepidermal water loss (TEWL) was measured with a TM300 connected to MPA5 (Courage + Khazaka, Cologne, Germany) and stratum corneum (SC) hydration was assessed with a Corneometer CM820 (Courage + Khazaka, Cologne, Germany). 2 days after the last treatment of DNCB, skin and blood samples were collected to evaluate the allergic inflammation and skin barrier function.

### 4.4. Measurement of Dermatitis Score and Epidermal Thickness

Atopic dermatitis severity and epidermal thickness were measured as described previously [69]. After mice were anesthetized with 2% isoflurane, skin lesions were photographed. Scores of 0 (none), 1 (mild), 2 (moderate), and 3 (severe) were given for each of the four symptoms: (i) erythema/hemorrhage, (ii) edema, (iii) excoriation/erosion and (iv) scaling/dryness. Maximum score is 12. Epidermal thickness of 10 randomly selected areas in three sections of each mouse was measured using a microscope (DM750; Leica, Wetzlar, Germany) aided by haematoxylin and eosin (H&E) staining and ImageJ version 1.51 (NIH, Bethesda, MD, USA). Assessments were performed in a blinded manner.

### 4.5. Measurement of Total Serum IgE and Inflammatory Cytokines

Blood collected from mice was placed in an e-tube and centrifuged at 4 °C and 850× *g* for 30 min. Following centrifugation, supernatant was stored at −70 °C until measurement. Serum IgE was measured using an enzyme-linked immunosorbent assay (ELISA). Each sample was added onto a 96-well plate treated with the primary antibody using a mouse IgE ELISA Kit (Invitrogen, Waltham, MA, USA), and washed four times with the working solution. After washing, the samples were treated with the secondary antibody, HRP-conjugated goat anti-mouse IgE (1:10,000), for 1 h after which a color development reagent was used. Following color development, the reaction was stopped using a stop solution, and absorbance was measured at 450 nm using a microplate reader (Spectra Max 250, Molecular Devices, Sunnyvale, CA, USA).

### 4.6. Histological Examination

Each tissue slide was prepared as described previously [69]. Staining was carried out via H&E and toluidine blue, followed by immunohistochemical staining with anti-filaggrin (Abcam, Cambridge, UK), anti-loricrin (Abcam), and anti-TRPA1 (GTX54765; GeneTex, Irvine, CA, USA) antibodies. Staining was performed using an Ultravision Quanto Detection System (TL-060-QHD; Thermo Fisher Scientific, Waltham, MA, USA). After staining, tissues were dehydrated and sealed in Permount (SP15-100; Thermo Fisher Scientific), and observed via optical microscopy (DM750, Leica, Wetzlar, Germany). For fluorescence images, the sections were incubated with primary antibody with anti-TRPA1 (GTX54765; GeneTex, Irvine, CA, USA) antibody at 4 °C for 16 h, followed by incubation with secondary antibodies against FITC-conjugated goat-anti-rabbit IgG (1:1000, sc-2012, Santa Cruz Biotechnology) at 21 °C for 1 h. Immunostained tissues were mounted with a medium containing Fluoroshield™ with DAPI (ImmunoBioScience, Mukilteo, WA, USA). Fluorescence images were acquired by using a confocal microscope (LSM700; Zeiss, Jena, Germany).

### 4.7. RNA Extraction and Real-Time PCR

Total RNA was extracted from isolated skin tissue using Tri-RNA reagent (Favorgen Biotech, Pingtung, Taiwan). Single strand cDNA synthesis from whole RNA templates was performed with PrimeScriptTM RT Master Mix (Takara, Tokyo, Japan). Resulting cDNA was subjected to real-time PCR with the CFX96 (Bio-Rad, Hercules, CA, USA) and qPCR 2 × PreMIX SYBR (Enzynomics, Seoul, Korea). PCR used to amplify all genes was performed for 40 cycles (95 °C for 10 min; 60 °C for 15 s; and 72 °C for 30 s) following the denaturation process at 95 °C for 10 min. Expression data were calculated as a cycle threshold (Ct) value using the ΔCt quantification method normalized to RPLP0. Oligonucleotides used for real-time PCR were as follows: mouse RPLP0, forward, 5′-GAT TCG GGA TAT GCT GTT GGC-3′, reverse, 5′-TCG GGT CCT AGA CCA GTG TTC-3′; mouse IL-4; forward, 5′-GGT CTC AAC CCC CAG CTA GT-3′, reverse, 5′-GCC GAT GAT CTC TCT CAA GTG AT-3′; mouse IL-13, forward, 5′-CCT GGC TCT TGC TTG CCT T-3′, reverse, 5′-GGT CTT GTG TGA TGT TGC TCA-3′; mouse IL-31, forward, 5′-TCA GCA GAC GAA TCA ATA CAG C-3′, reverse, 5′-TCG CTC AAC ACT TTG ACT TTC T-3′; mouse TSLP, forward, 5′-AGC TTG TCT CCT GAA AAT CGA G-3′, reverse, 5′-AGG TTT GAT TCA GGC AGA TGT T-3′; mouse RANTES(CCL5), forward, 5′-GCT GCT TTG CCT ACC TCT CC-3′, reverse, 5′-TCG AGT GAC AAA CAC GAC TGC-3′; mouse TARC(CCL17), forward, 5′-TAC CAT GAG GTC ACT TCA GAT GC-3′, reverse, 5′-GCA CTC TCG GCC TAC ATT GG-3′; mouse Keratin 1, forward, 5′-TGG GAG ATT TTC AGG AGG AGG-3′, reverse, 5′-GCC ACA CTC TTG GAG ATG CTC-3′; mouse Filaggrin, forward, 5′-ATG TCC GCT CTC CTG GAA AG-3′, reverse, 5′-TGG ATT CTT CAA GAC TGC CTG TA-3′; mouse Loricrin, forward, 5′-GCG GAT CGT CCC AAC AGT ATC-3′, reverse, 5′-TGA GAG GAG TAA TAG CCC CCT-3′; mouse Involucrin, forward, 5′-ATG TCC CAT CAA CAC ACA CTG-3′, reverse, 5′-TGG AGT TGG TTG CTT TGC TTG-3′; mouse TRPA1, forward, 5′-GTC CAG GGC GTT GTC TAT CG-3′, reverse, 5′-CGT GAT GCA GAG GAC AGA GAT-3′; mouse IL-31RA, forward, 5′-TCC TGA TGT TCC CAA CCC TG-3′, reverse, 5′-TTA GGA CCA CGT CTT CTG TGT-3′; mouse OSMR, forward, 5′-GAT TCG CAT CAC AGC CAA CAA-3′, reverse, 5′-CCA GAT ACG GGC TCC CAA GA-3′.

### 4.8. Western Blot Analysis

Cells and dorsal tissues were collected and lysed in PRO-PREP (iNtRON, Seongnam, Korea) containing a protease inhibitor cocktail (Complete™; Roche, Mannheim, Germany). Amounts of protein in the lysates were quantitated using a BCA kit (Thermo Fisher Scientific). Following quantitation, equal amounts of protein were resolved on 10% SDS-PAGE gels and electrotransferred to polyvinylidene fluoride membranes (Millipore, Bedford, MA, USA). After blocking with 5% skim milk in Tris-buffered saline containing 0.5% Tween-20 (TBST), membranes were probed with anti-filaggrin, anti-involucrin, anti-loricrin from Abcam (Cambridge, UK); anti-alix, anti-flotillin, anti-CD9, anti-phospho-STAT1, anti-STAT1, anti-phospho-STAT1, anti-STAT1, anti-phospho-STAT3, anti-STAT3 from Cell Singling Technology (Danvers, MA, USA); and anti-β-actin (Santa Cruz Biotechnology, Santa Cruz, CA, USA) antibodies at 4 °C overnight. After washing, membranes were incubated with HRP-conjugated anti-mouse (Vector Labs Inc., Burlingame, CA, USA) or anti-rabbit (Vector Labs Inc.) secondary antibodies. Immunoreactive signals were detected using enhanced chemiluminescence (ECL) reagents (EzWestLumi plus; ATTO, Tokyo, Japan).

### 4.9. Statistical Analysis

Continuous variables were tested for normal distribution by using the Shapiro-Wilk test. Data are presented as mean ± SD or as median [IQR] (min–max). Statistical analysis was performed by independent samples *t*-test for normally distributed data, or Mann-Whitney-U test was used. A value of *p* < 0.05 was considered statistically significant. All calculations were carried out by using SPSS Statistics 17 (SPSS, Chicago, IL, USA).

## 5. Conclusions

In conclusion, this study demonstrated the effect of cASCs and cASC-EVs in improvement of impaired skin in AD. cASCs and cASC-EVs positively regulated inflammatory responses and repair of defective epidermal permeability barrier functions by modulating gene expression, including epidermis differentiation and immune response. Furthermore, cASCs and cASC-EVs ameliorated pruritus by reducing IL-31/TRPA1 through the inhibition of JAK-STATs signaling activation. We further need to study the downregulation of STATs to clarify a more accurate relationship between IL-31/TRPA1 and STATs. These findings indicate that cASCs or cASC-EVs could be a novel therapeutic strategy to treat canine AD.

## Figures and Tables

**Table 1:** Statistical analysis I (parametric distribution).

Mean ± SD	Group 1 (Normal)	Group 2 (DNCB + Vehicle)	Group 3 (DNCB + EV)	Group 4 (DNCB + cASC)	*p*-Value * (Group 2–3)	*p*-Value * (Group 2–4)
Epidermal Thickness (Skin, μm)	23.8 ± 3.2	130.0 ± 23.9	88.4 ± 13.5	83.3 ± 20.8	0.002	0.003
Epidermal Thickness (Ear, μm)	13.7 ± 3.5	76.5 ± 36.8	37.0 ± 14.7	24.4 ± 3.9	0.044	0.018
Serum IgE (μg/mL)	42.4 ± 10.9	867.5 ± 194.6	588.9 ± 95.8	437.6 ± 123.6	0.016	0.002
Mast cell count (Skin, ea)	12.7 ± 5.3	57.0 ± 8.2	32.7 ± 12.4	34.7 ± 11.9	0.003	0.004
Mast cell count (Ear, ea)	11.7 ± 4.2	31.3 ± 4.2	20.8 ± 2.4	20.5 ± 2.3	<0.001	<0.001
IL-4 mRNA (fold)	1.0 ± 0.5	2.5 ± 1.4	1.4 ± 0.1	1.7 ± 0.2	0.094	0.197
IL-13 mRNA (fold)	1.0 ± 0.6	2.8 ± 1.3	1.3 ± 0.3	1.9 ± 0.6	0.039	0.163
IL-31 mRNA (fold)	1.0 ± 0.4	4.2 ± 2.2	1.0 ± 0.5	1.5 ± 0.5	0.016	0.030
TSLP mRNA (fold)	1.0 ± 0.4	5.6 ± 2.3	1.3 ± 1.1	0.6 ± 0.4	0.002	0.003
K1 mRNA (fold)	1.0 ± 0.2	0.6 ± 0.2	0.9 ± 0.1	1.0 ± 0.2	0.005	0.002
RANTES mRNA (fold)	1.0 ± 0.5	3.6 ± 1.1	1.6 ± 1.0	1.2 ± 0.3	0.007	0.002
TEWL (g/m^2^/h)	11.8 ± 2.0	39.7 ± 6.9	26.5 ± 3.6	22.5 ± 3.5	0.002	<0.001
Hydration (A.U.)	49.4 ± 5.6	33.5 ± 4.1	39.6 ± 7.9	41.4 ± 2.5	0.121	0.002
FLG mRNA (fold)	1 ± 0.1	0.5 ± 0.1	0.9 ± 0.3	0.6 ± 0.1	0.009	0.062
LOR mRNA (fold)	1 ± 0.3	0.6 ± 0.2	0.8 ± 0.1	0.8 ± 0.1	0.039	0.072
INV mRNA (fold)	1.0 ± 0.4	0.5 ± 0.2	0.7 ± 0.1	0.8 ± 0.2	0.068	0.063
TRPA1 mRNA (fold)	1.0 ± 0.4	2.1 ± 0.3	1.4 ± 0.1	1.5 ± 0.1	0.003	0.008
IL-31RA mRNA (fold)	1.0 ± 0.4	1.7 ± 0.4	1.3 ± 0.1	1.4 ± 0.1	0.038	0.128
OSMR mRNA (fold)	1.0 ± 0.6	3.3 ± 1.7	0.9 ± 0.5	0.6 ± 0.2	0.016	0.012
p-STAT1/STAT1 (fold)	1.0 ± 0.1	4.0 ± 0.4	0.7 ± 0.2	0.8 ± 0.2	<0.001	<0.001
p-STAT3/STAT3 (fold)	1.0 ± 0.1	3.1 ± 0.2	2.0 ± 0.5	1.3 ± 0.7	0.031	0.013

* Independent samples *t*-test.

**Table 2:** Statistical analysis II (nonparametric distribution).

Median [IQR] (Min–Max)	Group 1 (Normal)	Group 2 (DNCB + Vehicle)	Group 3 (DNCB + EV)	Group 4 (DNCB + cASC)	*p*-Value ** (Group 2–3)	*p*-Value ** (Group 2–4)
Dermatitis Score	0	8.5 [8.0–9.0] (3.0–10.0)	4.0 [4.0–4.0] (3.0–6.0)	3 [3.0–3.8] (3.0–4.0)	0.040	0.019
TARC mRNA (fold)	0.9 [0.8–1.2] (0.5–1.7)	5.0 [3.4–5.4] (1.8–8.3)	0.7 [0.6–1.4] (0.3–3.9)	1.8 [1.7–1.8] (1.6–1.9)	0.010	0.016

** Mann-Whitney U test.

## Data Availability

The data that support the findings of this study are available from the corresponding author upon reasonable request.
